# Attenuated total reflection FTIR dataset for identification of type 2 diabetes using saliva

**DOI:** 10.1016/j.csbj.2022.08.038

**Published:** 2022-08-20

**Authors:** Miguel Sanchez-Brito, Gustavo J. Vazquez-Zapien, Francisco J. Luna-Rosas, Ricardo Mendoza-Gonzalez, Julio C. Martinez-Romo, Monica M. Mata-Miranda

**Affiliations:** a Tecnológico Nacional de México, Campus Aguascalientes, Av. Adolfo López Mateos #1801 Ote, Fracc. Bona Gens, C.P. 20256 Aguascalientes, Ags., Mexico; b Instituto Politécnico Nacional, Escuela Superior de Cómputo, Av. Luis Enrique Erro S/N, Unidad Profesional Adolfo López Mateos, Zacatenco, Alcaldía Gustavo A. Madero, C.P. 07738 Ciudad de México, Mexico; c Escuela Militar de Medicina, Centro Militar de Ciencias de la Salud, Secretaría de la Defensa Nacional, Ciudad de México 11200, Mexico

**Keywords:** Attenuated total reflection FTIR, Dataset, Saliva, Diabetes, Glucose, A1C Test

## Abstract

Diabetes is one of the top 5 non-communicable diseases that occur worldwide according to the World Health Organization. Despite not being a fatal disease, a late diagnosis as well as poor control can cause a fatal outcome, because of that, several studies have been carried out with the aim of proposing additional techniques to the gold standard to assist in the diagnosis and control of this disease in a non-invasive way. Considering the above, and in order to provide a solid starting point for future researches, we share a primary research dataset with 1040 saliva samples obtained by Fourier Transform Infrared Spectroscopy considering the Attenuated Total Reflectance method. Database include: gender, age, individuals (patients) with/without diabetes, the glucose value, and the result to the A1C test for the diabetic population. We believe that sharing dataset as is could increase experimentation, research, and analysis of spectra through different strategies broaden its range of applicability by chemists, doctors, physicists, computer scientists, among others, to identify the effects that the virus causes in the body and to propose possible clinical treatments as well as to develop devices that allow us to assist in the characterization of possible carriers.

## 1 Introduction.

According to the World Health Organization (WHO), non-communicable diseases (NCDs) claim the lives of approximately 41 million people each year. NCDs, also known as chronic diseases, tend to be long-lasting and result from a combination of genetic, physiological, environmental, and behavioral factors. The main types of NCDs are cardiovascular diseases (such as heart attacks and strokes), cancer, chronic respiratory diseases (such as chronic obstructive pulmonary disease and asthma), and diabetes. Regarding diabetes, the WHO notes that around 95 % of people with diabetes have ineffective use of insulin by the body, this type of diabetes is known as type 2 diabetes. Despite not being a fatal disease, risks like the increased probability of suffering a heart attack compared to people who do not suffer from it, neuropathies, diabetic retinopathy or kidney damage by patients who suffer from it, can lead to death if the necessary measures are not taken to monitor the variations in glucose levels in the body that are characteristic of this disease.

Despite the existence of reliable strategies and devices for both the diagnosis and control of this disease, it has not been possible to reduce the mortality rate associated with this pathology, thus, assistance in the clinical diagnosis of pathologies in a non-invasive way is currently a topic that is addressed through different techniques. Fourier Transform Infrared Spectroscopy (FTIR) is commonly used due to the nature of the samples analyzed. Through the interaction of a biological sample with electromagnetic frequencies associated with the middle region of the infrared spectrum expressed as wavenumbers (cm^−1^), it is possible to produce vibrations in the chemical bonds that make up the sample [1], [2]. The wavenumbers considered in the mid-infrared region range between 400 and 4000 cm^−1^ [1], [2], [3], [4], [5]. Once the interaction of the sample with these wavenumbers has ended, the vibrations are captured in a two-dimensional matrix called FTIR spectrum.

The search for a spectral behavior attributed to a population with a specific pathology in common could be the key to assisting in clinical diagnosis through FTIR spectroscopy. However, the more elements that make up the sample that is analyzed, the more complicated it will be to detect a spectral behavior typical of pathology due to the overlapping of the vibrations of the elements that make it up [1], [2], [6], [10], [11]. According to [3], [4], [6], [7], [8], [9], [10], the highest vibrations recorded in a biological sample belong mainly to carbohydrates and nucleic acids (1225 cm^−1^), lipids (1750 cm^−1^) and proteins (amide I: 1550–1600 cm^−1^ and amide II: 1600–1700 cm^−1^ mainly). Punctually, research related to diabetes mainly highlights the differences against the control group specifically in the region of lipids and carbohydrates and nucleic acids [7], [8], [9], [10]. Although the above helps to identify the most contrasting spectral regions of the spectra that make up the studies of the different authors, it is not enough to propose a strategy that allows a spectrum to be reliably identified as suggested by the validation metrics reported by the authors [7], [8] due to the overlapping of the spectra.

In this sense, multivariate analysis techniques commonly used in the area of machine learning have allowed better results, compared to techniques such as principal components (PCA) as reported by the authors of [10], [12], [13], [14], however, these strategies are not infallible, their effectiveness is closely related to the number of samples that the model can analyze to identify patterns in the populations studied, and this is a field of study currently open to research. In order to provide a starting point to the scientific community that wishes to direct its efforts to find patterns in the FTIR spectra of saliva samples, we share our database made up of 1040 patients, of which 500 formed the control group and the rest belongs to patients previously confirmed with diabetes through the gold standard strategies. The database includes the gender and age of the patient from whom the saliva sample was obtained and, in the case of diabetic patients, the glucose values and the A1C test result are provided.

## 2 Materials and methods.

Between February 2019 and February 2020, saliva samples belonging to 1040 patients were collected under informed consent and following the guidelines outlined in the Declaration of Helsinki to protect the information of patients participating in clinical research projects. The patients were confirmed with and without type 2 diabetes in the Unidad de Especialidades Médicas (UEM) of the Secretaria de la Defensa Nacional (SEDENA, Mexico) after having approved the internal research protocol with folio: 001/2019. All experiments were examined and approved by the appropriate ethics committee and followed the ethical standards laid down in the 1964 Declaration of Helsinki.

The inclusion criteria set for obtaining the saliva sample from patients were:

• Patients between the ages of 20 and 80 gave their consent to provide a sample of approximately 1 ml of saliva in a micro-centrifuge tube previously sterilized.
• The patients had to be in a fasting period of at least 8 h.
• They should not have brushed their teeth or used mouthwash; likewise, they should not have had any orthodontic treatment or any treatment in the oral cavity.
• Selected patients should not wear any type of lipstick.
• The patients considered as a control group involve different pathologies but no type of diabetes.

Every day between 10 and 15 samples were collected in a period no longer than 2 h to avoid sample degradation as much as possible. The patients considered in the present study were sampled between 6 and 8 in the morning. All the collected samples were refrigerated at a temperature between 0 °C and 4 °C and processed after the 2 h defined for the sampling process [3], [6], [11]. The saliva sample was obtained through the process of spitting by the patient into the micro-centrifuge tube, previously they were instructed to try not to stimulate the production of the same fluid. The micro-centrifuge tube was handled only by the doctor on duty using nitrile gloves each time each sample was obtained and processed.

The Attenuated Total Reflection (ATR) technique was used to capture the absorbance spectrum through a Jasco FTIR-6600 spectrometer located at the Escuela Militar de Medicina (EMM) of the SEDENA. From each saliva sample, 3 μl were deposited by pipetting directly on the crystal of the equipment. Plastic tips previously sterilized were used in the pipette to obtain the mentioned amount of sample, which were discarded after placing each sample. To speed up the sample drying process, the environment temperature was increased by turning on incandescent lamps for 15 min, since after that time it was possible to appreciate the main macromolecular groups reported by [3], [4], [5], [6], [7]. Due to the large amount of water molecules with respect to others in biological samples, it has been one of the main challenges in the study of this type of specimens using FTIR spectroscopy as pointed out by [15], [16], so the drying process is essential to appreciate the main macromolecules mentioned. At the time of taking the sample, the lamps were deactivated so that the radiation they could emit would interfere with the measurement. The configuration used for the capture was 120 scans with a resolution of 4 cm^−1^ as suggested by [1], [2], [6]. After the drying process and once the spectrum has been captured, using the Spectra Manager version 2 software, included with the spectrometer, the noise reduction processes by H_2_O, CO_2_, and baseline adjustment were performed. In addition to the saliva sample, gender and age were recorded.

## 3 Results

The 1040 spectra were grouped in a.csv extension file according to the following distribution:

**Column 1:** Gender,

**Column 2:** Age,

**Column 3:** Population (DIABETES, CONTROL),

**Column 4 to 3739:** Absorbance values obtained with wavenumbers between 399 and 4000 cm^−1^.

Information on the study population is presented in Table 1.

**Table 1:** Analyzed population.

Group	Gender	Total patients	Age (Mean) years	Total patients by age ranges	
				Age range (years)	Total patients
Type 2 diabetes	FEMALE	336	60.7±11.43	20–29	3
				30–39	10
				40–49	36
				50–59	99
				60–69	107
				70–79	68
				80–89	13
	MALE	204	61.11±11.54	20–29	2
				30–39	3
				40–49	31
				50–59	45
				60–69	76
				70–79	44
				80–89	3

Control	FEMALE	328	52.57±14.52	20–29	19
				30–39	38
				40–49	77
				50–59	102
				60–69	52
				70–79	29
				80–89	11
	MALE	172	53.66±14.10	20–29	8
				30–39	24
				40–49	31
				50–59	48
				60–69	32
				70–79	26
				80–89	3

**Fig. 1:**
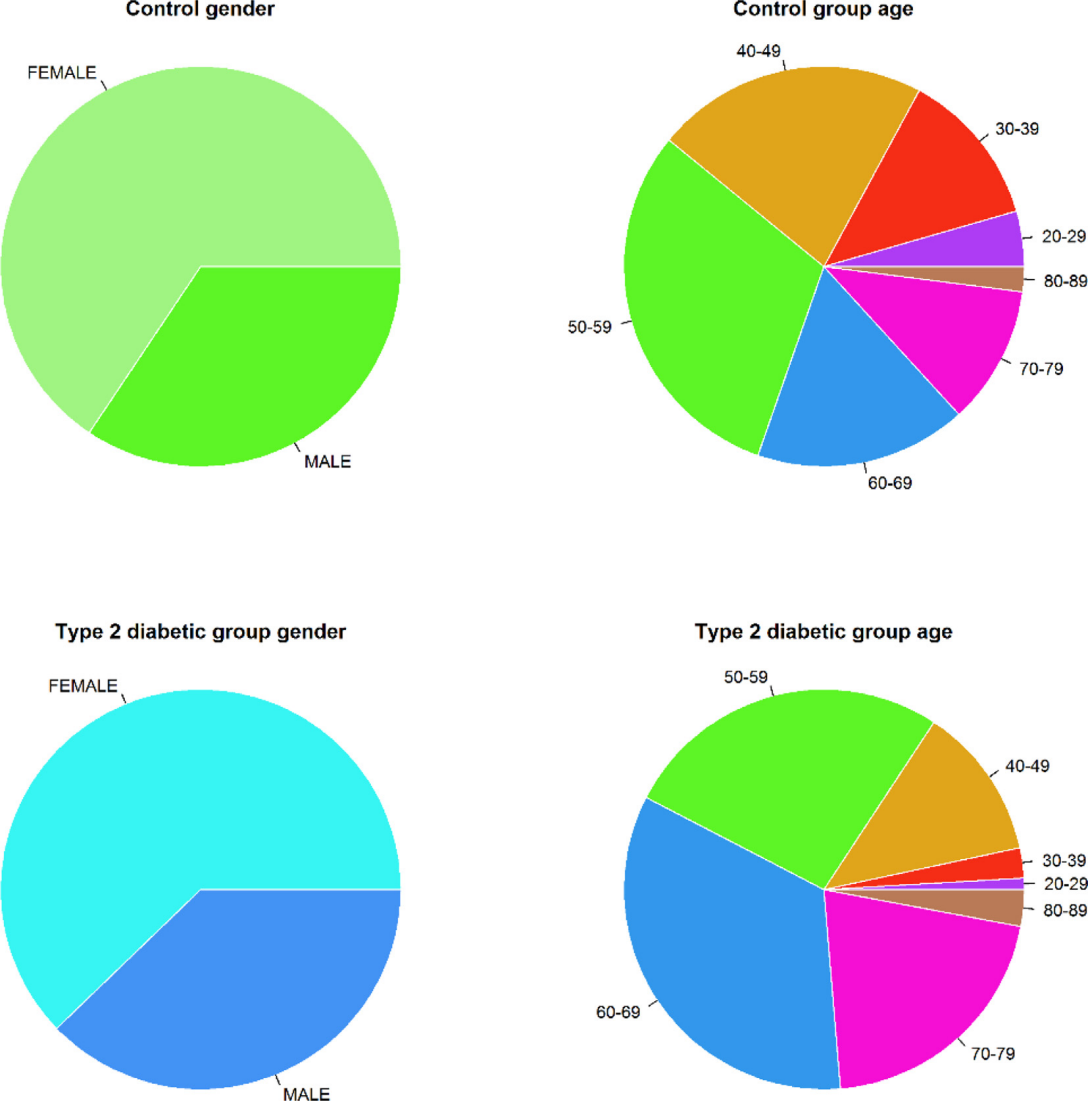
Gender and age distribution of the groups studied.

Gender and age distributions of the diabetic/control analyzed population are graphically depicted in Fig. 1.

Additionally, Table 2 and Table 3 summarizes the glucose and hemoglobin values, respectively. These percentages were obtained through the A1C test from patients previously diagnosed with type 2 diabetes.

**Table 2:** Glucose values information.

Population	Group	Total samples	Glucose range (mg/dl)	Range mean (mg/dl)
Type 2 diabetes	1	114	[45–100)	85.6
	2	217	[100–150)	121.9
	3	99	[150–200)	172.3
	4	60	[200–250)	223.5
	5	26	[250–300)	272.5
	6	24	>300	344.7

**Table 3:** Hemoglobin test A1C Test information.

Population	Group	Total samples	A1C Test (%)	Range mean (%)
Type 2 diabetes	1	3	[4–5)	4.7
	2	61	[5–6)	5.6
	3	116	[6–7)	6.4
	4	89	[7–8)	7.4
	5	78	[8–9)	8.3
	6	47	[9–10)	9.3
	7	106	>10	11.3

**Fig. 2:**
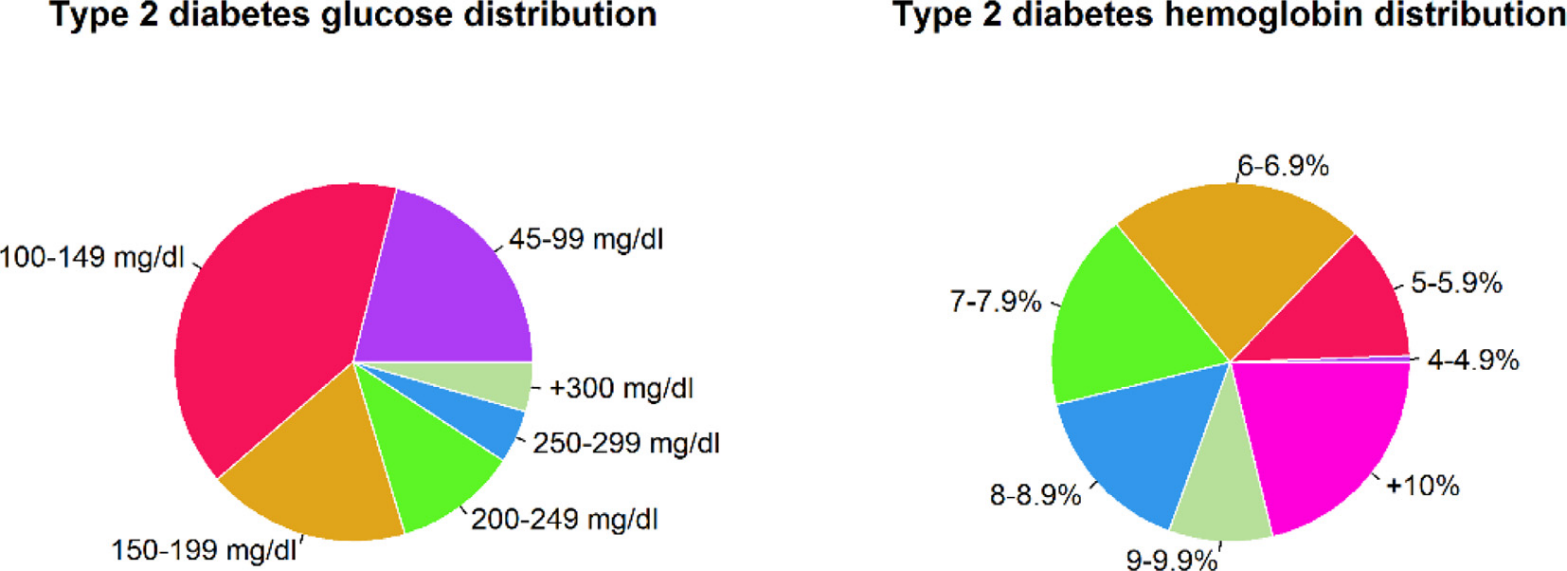
Distribution of glucose and hemoglobin values recorded for diabetic patients.

In Fig. 2, the distribution of glucose (mg/dl) and hemoglobin (%) values recorded in the patient clinical history log when collecting the saliva sample is presented.

**Fig. 3:**
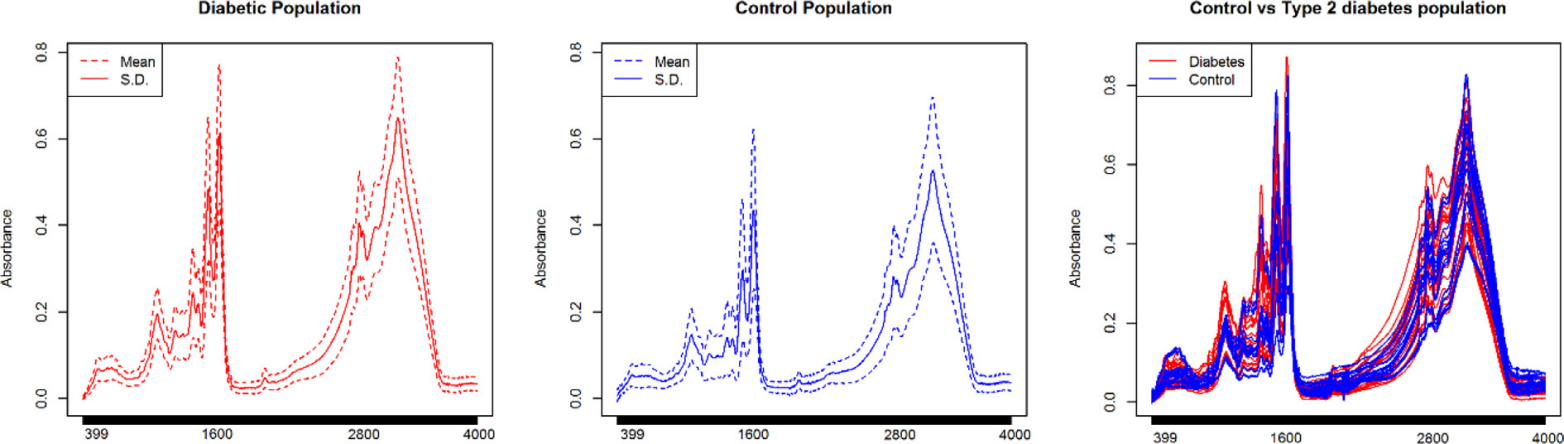
Mean spectra of populations with and without type 2 diabetes (red and blue, respectively). The dotted lines involve adding and subtracting one standard deviation from the mean. In the third figure, 20 spectra of the control group (blue) are contrasted against 20 spectra of the diabetes group (red). (For interpretation of the references to colour in this figure legend, the reader is referred to the web version of this article.)

In works such as those of [6], [12], [13], the difficulty of selecting a characterization technique has been pointed out, having to experiment with several of them to adapt the one that allows obtaining the best results. Comparing the measures of central tendency of two or more populations could help delimit the selection of characterization strategies, if in addition to the above, more information on the behavior of the signals is provided, the selection process of characterization techniques can be optimized. In this sense we present Fig. 3, where the average spectrum of the population previously diagnosed with type 2 diabetes as a solid red line and the average of the control group population in blue. The dotted lines involve the addition and subtraction of one standard deviation (S.D.) from the mean spectrum of each population. For example, the figure titled *Control vs Type 2 diabetes population* presents 20 spectra of the control group and 20 of the group previously diagnosed with type 2 diabetes; it is possible to appreciate the complexity of using FTIR spectroscopy to assist in the diagnosis of any pathology using a sample with a large number of components such as saliva.

The addition and subtraction of a standard deviation from the mean allows knowing the behavior of most of the signals (>90 %) recorded for a given population. In this way it would be possible to identify regions of the spectrum of a certain population that could be associated with a certain pathology if it presents a small standard deviation [13]. Likewise, it would be possible to select or avoid experimentation with clustering strategies based on the calculation of distances between spectra such as K-means, since they would be affected by the overlap of the signals.

**Fig. 4:**
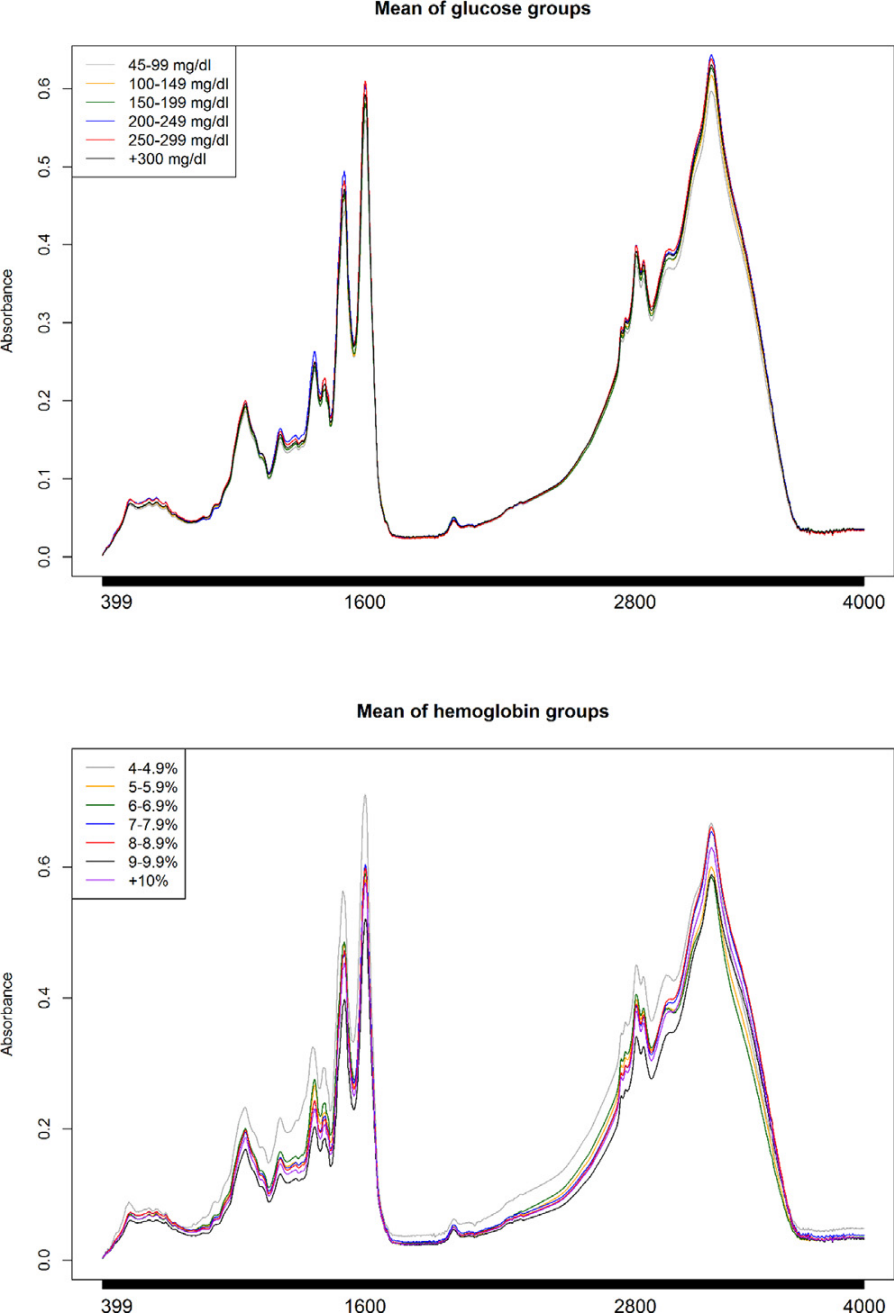
Behavior of the means of the glucose and hemoglobin groups.

Fig. 4 shows the behavior of the means of the glucose and hemoglobin groups according to [6], [11], the analysis of only the region between (900–1900 cm^−1^) called biological fingerprint (BFP) is common because vibrations of a large number of molecules have been reported in this region: carbohydrates, proteins, lipids, and deoxyribonucleic acid (DNA), mainly, however, it is possible to appreciate that for the means of the glucose groups, the similarity in absorbance values could make it difficult to adopt a strategy that allows characterizing FTIR spectra of saliva samples based on the glucose values of the patients, not so for the means of the groups of hemoglobin values, where a greater separation in the groups is observed.

A region commonly omitted in the analysis of biological samples is the one between the wavenumbers 2800 and 3700 cm^−1^ approximately. This is due to the vibrations associated with hydrogen bonds, since it is the main constituent of saliva, the high content of bonds with this element would hide the other vibrations of the different molecular groups. However, in this region, important vibrations with lipids and proteins (amide A) have been reported [3], [5], [11]; these regions could be helpful to record the N or O glycosylation process [17], [18], [19], [20].

In the 2800–3700 cm^−1^ region, mainly for the means of the glucose groups, a greater dispersion in the absorbance values is observed compared to the BFP region, which could help in the characterization of FTIR spectra of saliva samples, both for diabetic patients and to propose a strategy that allows associating reliably the spectrum with a given glucose value the vibrations of these molecules have also been highlighted by [21]. However, the authors consider the vibrations recorded in the wavenumbers 1462–1747 cm^−1^ since their study focuses on the analysis of the BFP.

## 4 Discussion

According to the World Health Organization (WHO), diabetes is one of the top 5 non-communicable diseases that cause the most deaths worldwide, with type 2 diabetes being the most common. Despite not being a fatal disease, the deterioration in the health of patients who suffer from it can lead to death if the necessary measures are not taken to monitor the variations in glucose levels in the body that are characteristic of this disease [27].

In order to assist in the diagnosis and control of this disease, numerous organizations have developed commercial devices that allow people with this disease to monitor the sugar levels in their bodies. Despite this, the rate of deaths reported each year due to health complications associated with this disease has not decreased according to WHO figures. The foregoing could be attributed to different factors such as: the patient's own response, lack of time to perform physical activity, lack of knowledge of the correct diet that a diagnosed patient should follow, discrepancy between the measurements of the different devices to monitor the levels of sugar in the body, etc. In addition to the above, the bio fluid that the devices analyze to estimate the percentage of sugar in the body could be associated: the blood. The need to analyze blood to estimate the level of sugar in patients, although it is the most reliable way to make such an estimate, involves people suffering from this disease having to extract the body fluid (usually through a finger prick), this implies that in addition to the acquisition of the device that will carry out the analysis and display the result to the patient, the need to consider both, the equipment that allows causing the wound to extract the blood (lancets) and the means to conduct the blood to the device (reactive strips), because these consumables can only be used once, the economic investment in the short and medium term could be considerable for most patients, regardless of the physical discomfort it causes.

The blood study is mainly due to the analysis of hemoglobin. Hemoglobin, a protein that links up with glucose, is found inside red blood cells. Its job is to carry oxygen from the lungs to all the cells of the body. Glucose enters your red blood cells and links up (or glycates) with molecules of hemoglobin. The more glucose in your blood, the more hemoglobin gets glycated. By measuring the percentage of A1C in the blood, you get an overview of your average blood glucose control for the past few months [28]. Although hemoglobin is exclusive to the bloodstream, various studies have already confirmed the presence of both, glucose and glycoproteins similar to hemoglobin in different biofluids such as saliva [19], [20]. Considering biofluids that are non-invasive extraction would not only eliminate physical discomfort for patients, but would also allow them to avoid the need to purchase consumables to monitor their glucose levels, however, a separate case is the strategy with which the sample will be analyzed.

FTIR spectroscopy makes it possible to obtain a map of the sample's chemical structure that is analyzed thanks to the interaction of the bonds of the molecules that make it up with electromagnetic frequencies belonging to the middle region of the infrared spectrum. By analyzing the structure of samples of two or more populations, it would be possible to find a mathematical model that would allow them to be reliably characterized. Some of the studies that have shown the feasibility of using FTIR spectroscopy to assist in the diagnosis and control of diabetes are those presented by [3], [6], [7], [8], [9], [10], [14], [15], [16], [17], [18], [19], [20], [21], [22], [23], [24], [25], [26], [27], [28], [29], [30], [31], where the problem of overlap between the spectra of the studied populations is reduced thanks to machine learning (ML) techniques such as those suggested by [4], [5], [6], [22], [23], [24], [25], [26].

The studies mentioned, despite the good results reported, do not allow us to think about the development of a strategy that, through FTIR spectroscopy and using ML, allows us to reliably carry out the diagnosis and control of diabetic patients. The foregoing due to the need for a larger population as well as the processing of the samples considering different equipment but the same calibration parameters. In this way ML techniques could reliably identify patterns unique to diabetes in FTIR spectra.

With the present work, we make available to the public, the signals obtained by Fourier transform infrared spectroscopy of saliva samples from 1040 patients, of which 500 belong to the control group, and 540 belong to the group of patients diagnosed with type 2 diabetes in advance. In addition to the classes to which each signal belongs, for the population diagnosed with type 2 diabetes, the values obtained for glucose and the percentage obtained by the patient for the hemoglobin A1C test are provided. The shared spectra do not contemplate any normalization, smoothing, or transformation processing except indicated in the Materials and Methods section to provide a blank framework for experimentation. The shared spectra could be used to develop additional strategies to those proposed in the current state of the art and assist in the diagnosis and control of diabetes or be used as a control group to identify differences against other pathologies.

## 5 Conclusion

According to the World Health Organization (WHO), diabetes is one of the top 5 non-communicable diseases that cause the most deaths globally [27], with type 2 diabetes being the one that most affects the population.

The present work provides a compilation of FTIR spectra of saliva samples from people with and without type 2 diabetes. The database consists of 1040 samples 540 spectra of patients previously diagnosed with type 2 diabetes and 500 confirmed patients without this condition to provide a solid base to evaluate methodologies that allow to assist in the diagnosis of this pathology in a non-invasive way and complement with spectra of different populations, since according to an initial inspection of the distribution of the absorbance values of the spectra, its dispersion makes it difficult to define a region that reliably allows the characterization of the populations.

We fully agree with the fact that in order to analyzing FTIR spectra of saliva samples to assist in the diagnosis and control of diabetes in which useful regions are indicated for the characterization of the populations’ spectra it is necessary to carry out certain pre-processing such as normalization and noise reduction (including standard normal variate, Savitzky-Golay filter, among others) in the signals before comparing them. Nevertheless, we also believe that sharing and reusing primary research datasets in this case, spectra without any pre-processing except for that carried out by the FTIR spectrometer itself (the noise reduction processes by H_2_O, CO_2_, and baseline adjustment)– may foster experimentation, research, and analysis of spectra through different strategies reaching a broader community of researchers as the authors of [28] suggest when sharing databases.

Although it does not always happen, most patients with type 2 diabetes also have additional health disorders such as obesity, hypertension, hyperlipidemia, kidney diseases and cardiovascular disease, among others [29], [30], [31], so having the metrics associated with these ailments would be desirable. The absence of the metrics of the disorders mentioned above in the database are a limitation to reinforce the relationship between type 2 diabetes with these diseases, however, they are not a limitation to carry out associated studies for both diagnosis and monitoring of glucose levels in the human body analyzing saliva.

Finally, all spectra reported in this paper are also available in editable and implementable format at https://doi.org/10.6084/m9.figshare.19450916.v1.

## Declaration of Competing Interest

The authors declare that they have no known competing financial interests or personal relationships that could have appeared to influence the work reported in this paper.
